# Lacrimal gland excision in male and female mice causes ocular pain and anxiety-like behaviors

**DOI:** 10.1038/s41598-020-73945-w

**Published:** 2020-10-14

**Authors:** Neal E. Mecum, Danielle Demers, Cara E. Sullivan, Tori E. Denis, John R. Kalliel, Ian D. Meng

**Affiliations:** 1 Center for Excellence in the Neuroscience, University of New England, Biddeford, ME 04005 USA; 2 Molecular and Biomedical Sciences, University of Maine, Orono, ME 04469 USA; 3 Graduate Studies in Biomedical Sciences and Engineering, University of Maine, Orono, ME 04469 USA; 4 Department of Biomedical Sciences, College of Osteopathic Medicine, University of New England, Biddeford, ME 04005 USA

**Keywords:** Peripheral nervous system, Sensory processing, Chronic pain

## Abstract

Lacrimal gland excision (LGE) induced dry eye produces more severe corneal damage in female mice, yet signs of LGE-induced ocular pain and anxiety in male and female mice have not been characterized. Excision of either the extraorbital gland (single LGE), or both the extraorbital and intraorbital glands (double LGE) was performed in male and female C57BL/6J mice to induce moderate and severe dry eye. Ongoing pain was assessed by quantifying palpebral opening and evoked nociceptive responses after corneal application of capsaicin and menthol. The open-field and plus maze were used to assess anxiety. Single LGE caused a reduction in palpebral opening and an increase in capsaicin and menthol-evoked responses only in female mice. Furthermore, single LGE produced signs of increased anxiety in female but not male mice. Overall, female mice appear more susceptible to signs of ocular pain, irritation, and anxiety in response to aqueous tear deficiency.

## Introduction

Dry eye disease (DED) is marked by the sensation of ocular dryness and irritation, information conveyed to the brainstem through the activation of corneal primary afferent neurons^1^. The ability of corneal afferents to sense potentially damaging stimuli and monitor ocular dryness protects the eye and regulates the ocular tear film by controlling reflexive secretions and blinking^2,3^. Additional signs of DED include a reduction in tear volume, tear film instability, increased tear osmolarity, inflammation and damage to the ocular surface, yet the sensation of ocular pain is perhaps the most consistent finding, which if left untreated may lead to non-ocular comorbidities such as anxiety and depression^4–7^.

Aqueous tear deficiency, a form of dry eye caused by insufficient tear production from the lacrimal glands, has been modeled in the rodent through excision or functional disruption of the lacrimal glands^8–14^. Lacrimal gland excision (LGE) has been demonstrated to sensitize corneal polymodal nociceptive neurons to the TRPV1 agonist capsaicin and low pH induced by CO_2_, as well as sensitize corneal cold cells to the TRPM8 agonist menthol^11,15–17^. Furthermore, LGE-induced plasticity in corneal afferents includes an increase in co-expression of TRPM8 and TRPV1, which is matched by a greater proportion of cold responsive corneal afferents that are activated by noxious heat after LGE^18^.

The sensitization of polymodal corneal afferents is consistent with the increased eye wipe response observed after LGE in response to hypertonic saline in male rats^11,15^. In addition, unilateral LGE increased mechanical sensitivity and spontaneous blinking in the ipsilateral compared to contralateral eye in male rats. Of note, the increase in spontaneous blinks, a possible sign of ongoing ocular pain, was reduced by the topical anesthetic proparacaine^11^. In the guinea pig, LGE also increased blinking evoked by a low concentration of menthol^16^.

A recent comparison of female and male rats found that LGE resulted in greater signs of corneal damage and produced a greater increase in blink rates in female rats^13^. Consistent with these results, a significant sex difference has been reported in the inflammatory response to LGE in male and female mice. Despite producing a comparable reduction in tear volume in male and female mice, LGE caused greater immune cell infiltration and corneal epithelial cell apoptosis in female compared to male mice^10^. These results are consistent with epidemiological reports that DED is more prevalent in women compared to men^19–22^.

In a mouse model of corneal injury, sex differences have also been reported in nerve regeneration and wound healing. Following corneal epithelial cell debridement, female mice had a faster rate of nerve regeneration compared to males, although most strains of female mice showed a slower rate of healing^23^. The present study compared corneal sensitivity and innervation in male and female mice using LGE to produce chronic aqueous tear deficiency. Given the high comorbidity of anxiety with DED, as well as the higher levels of fear induced by pain of trigeminal origin^24^, the ability of LGE to affect anxiety related behaviors was also examined.

## Results

### Lacrimal gland excision reduces palpebral opening

**Figure 1:**
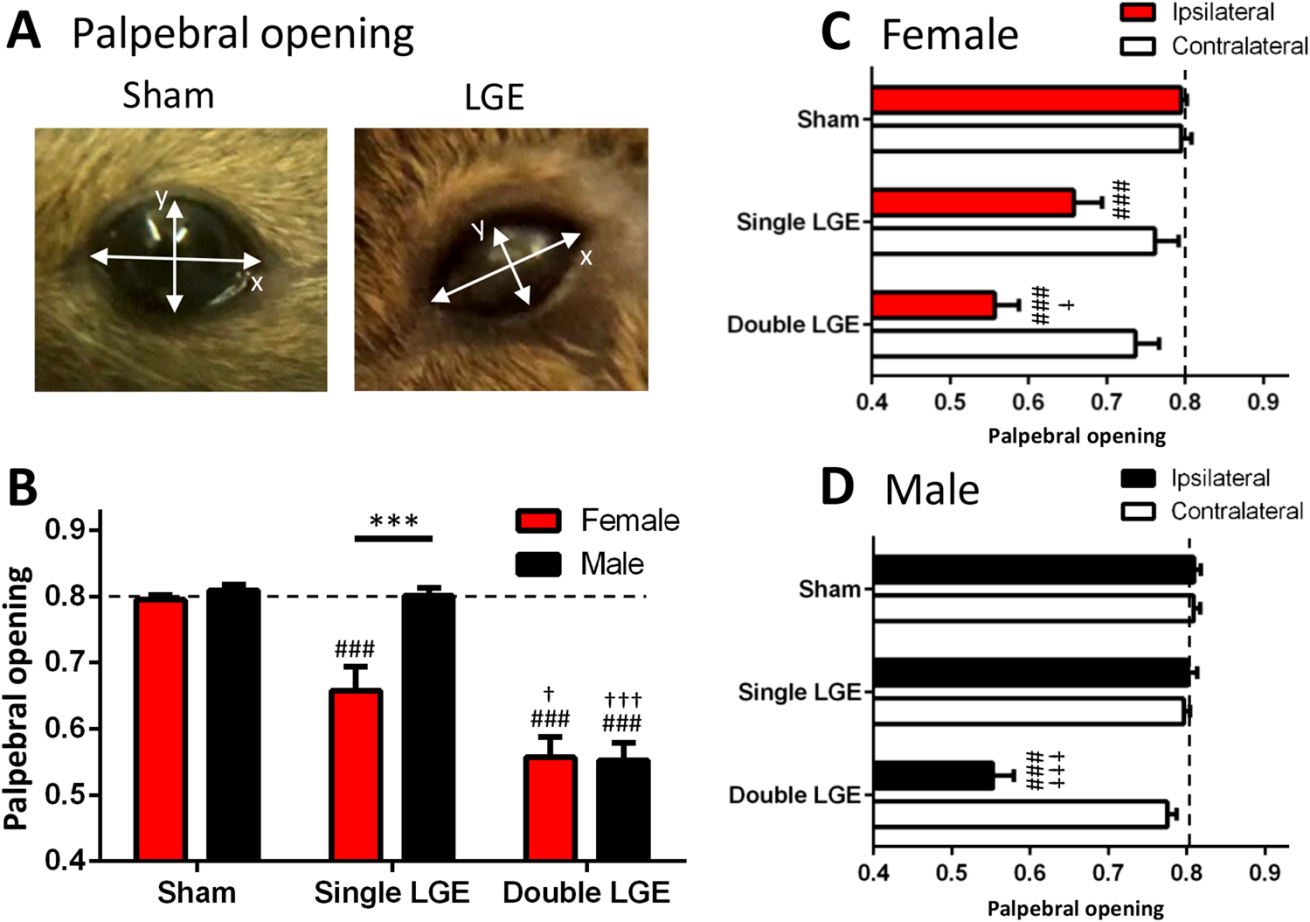
Palpebral opening following lacrimal gland excision in female and male mice. (**A**) Representative photos showing an eye from a sham animal (left) and from an animal following double LGE (right). The palpebral opening was calculated as the ratio Y/X. (**B**) Single LGE caused a significant reduction in palpebral opening only in female mice, whereas double LGE produced comparable reductions in the palpebral opening between female and male mice. (**C**) In female mice, both single and double LGE resulted in a significant reduction in palpebral opening in the eye ipsilateral to gland excision. (**D**)In male mice, only double LGE caused a significant reduction in palpebral opening on the side ipsilateral to gland excision. Dashed line represents naïve palpebral opening score. n = 12/treatment group. *p < 0.05, **p < 0.01, ***p < 0.001. ^###^p < 0.001 compared to sham of the same sex; ^†^p < 0.05, ^†††^p < 0.001 compared to single LGE of the same sex.

Previous studies in rats showed that LGE increased the rate of blinking ipsilateral to the side of excision^11^. In the mouse, however, clear blinking behavior was difficult to quantify since the response to LGE was to squint rather than blink. In order to quantify this squinting behavior, palpebral opening was measured by using a ratio consisting of the height of the gap between the upper and lower eyelids and the distance separating the two canthi (Fig. 1A). Using naïve male and female C57BL/6J mice, the value for the palpebral opening ratio was determined to be 0.801 ± 0.01 (n = 8), denoted as a dashed line in Fig. 1B.

An examination of palpebral opening two-weeks after LGE in female and male animals found a significant effect of both sex and surgery (2-way ANOVA, Table 1). Sham surgery did not affect the mean palpebral opening in female and male mice (0.794 ± 0.007 and 0.809 ± 0.008, respectively, Fig. 1B), which was similar to values recorded in naïve animals. Post hoc analysis revealed a difference between female and male mice after single LGE (0.657 ± 0.036 versus 0.801 ± 0.011, p < 0.001, Fig. 1B), whereas double LGE produced a comparable reduction in palpebral opening in both female and male mice (0.556 ± 0.03 and 0.552 ± 0.024, respectively, Fig. 1B). Furthermore, following single LGE, female mice had a significant reduction in palpebral opening compared to sham treated mice (Fig. 1B, p < 0.001), whereas palpebral opening in male mice did not differ from sham controls (Fig. 1B, p  > 0.05).

**Table 1:** Results from two-way ANOVA statistical analysis for all data sets.

Figure #	Measurement (units)	Sex (male/female)			Surgery			Interaction		
		dfn, dfd	F	p	dfn, dfd	F	p	dfn, dfd	F	p
Figure 1B	Palpebral opening	1, 66	7.224	< 0.0091	2, 66	59.69	< 0.0001	2, 66	5.977	< 0.0041
Figure 3A	Eye wipe	1, 64	7.674	< 0.0073	2, 64	3.074	< 0.0473	2, 64	4.039	< 0.00223
Figure 3B	Palpebral opening	1, 66	5.539	< 0.0216	2, 66	3.687	< 0.0304	2, 66	1.989	< 0.1449
Figure 5B	Time open arms (%)	1, 93	9.433	< 0.0028	2, 93	11.63	< 0.0001	2, 93	1.843	< 0.1640
Figure 5C	Time closed arms (%)	1, 93	6.863	< 0.0103	2, 93	19.3	< 0.0001	2, 93	1.718	< 0.1851
Figure 6B	Sholl intersections (subbasal)	1, 24	1.681	< 0.2071	1, 24	3.092	< 0.0914	1, 24	0.1292	< 0.7225
Figure 6B	Sholl intersections (terminals)	1, 24	0.2858	< 0.5978	1, 24	1.171	< 0.2900	1, 24	0.2024	< 0.6568
Figure 6C	Pixel density (subbasal)	1, 24	3.842	< 0.0648	1, 24	0.6278	< 0.4379	1, 24	2.072	< 0.1663
Figure 6C	Pixel density (terminals)	1, 24	0.002819	< 0.9581	1, 24	0.8080	< 0.3777	1, 24	0.1608	< 0.6920
Figure 6D	Tears (mm)	1, 33	1.303	< 0.2619	1, 33	137.1	< 0.0001	1, 33	0.0398	< 0.8429

		Side (ipsi/contra)			Surgery			Interaction		
Figure 1C	Palpebral opening	1, 66	18.65	< 0.0001	2, 66	15.546	< 0.0001	2, 66	5.639	< 0.0055
Figure 1D	Palpebral opening	1, 66	38.97	< 0.0001	2, 66	64.81	< 0.0001	2, 66	41.81	< 0.0001

The two independent factors included surgery (sham, single lacrimal gland excision, double lacrimal gland excision) and either sex (male, female) or side (ipsi- or contralateral to side of surgery)

*Dfn* degrees of freedom numerator, *dfd* degrees of freedom denominator, *F* F statistic, *p* p-value.

A comparison of the palpebral opening between the eye ipsilateral to surgery and the contralateral eye indicated a significant effect of both surgery treatment and side (ipsi/contra) in female and male mice (2-way ANOVA, Table 1). On the side contralateral to surgery, the palpebral opening was similar between sham controls, single LGE, and double LGE in both female and male mice (Fig. 1C,D). In the ipsilateral eye, single and double LGE caused a significant reduction in palpebral opening in female mice when compared to the contralateral eye (Fig. 1C). In contrast, only double LGE reduced the palpebral opening in male mice when compared to the contralateral eye (Fig. 1D). Double LGE also produced a greater reduction in palpebral opening when compared to single LGE in both female and male mice (Fig. 1C,D).

### Topical anesthetics reverse the effect of LGE on palpebral opening

**Figure 2:**
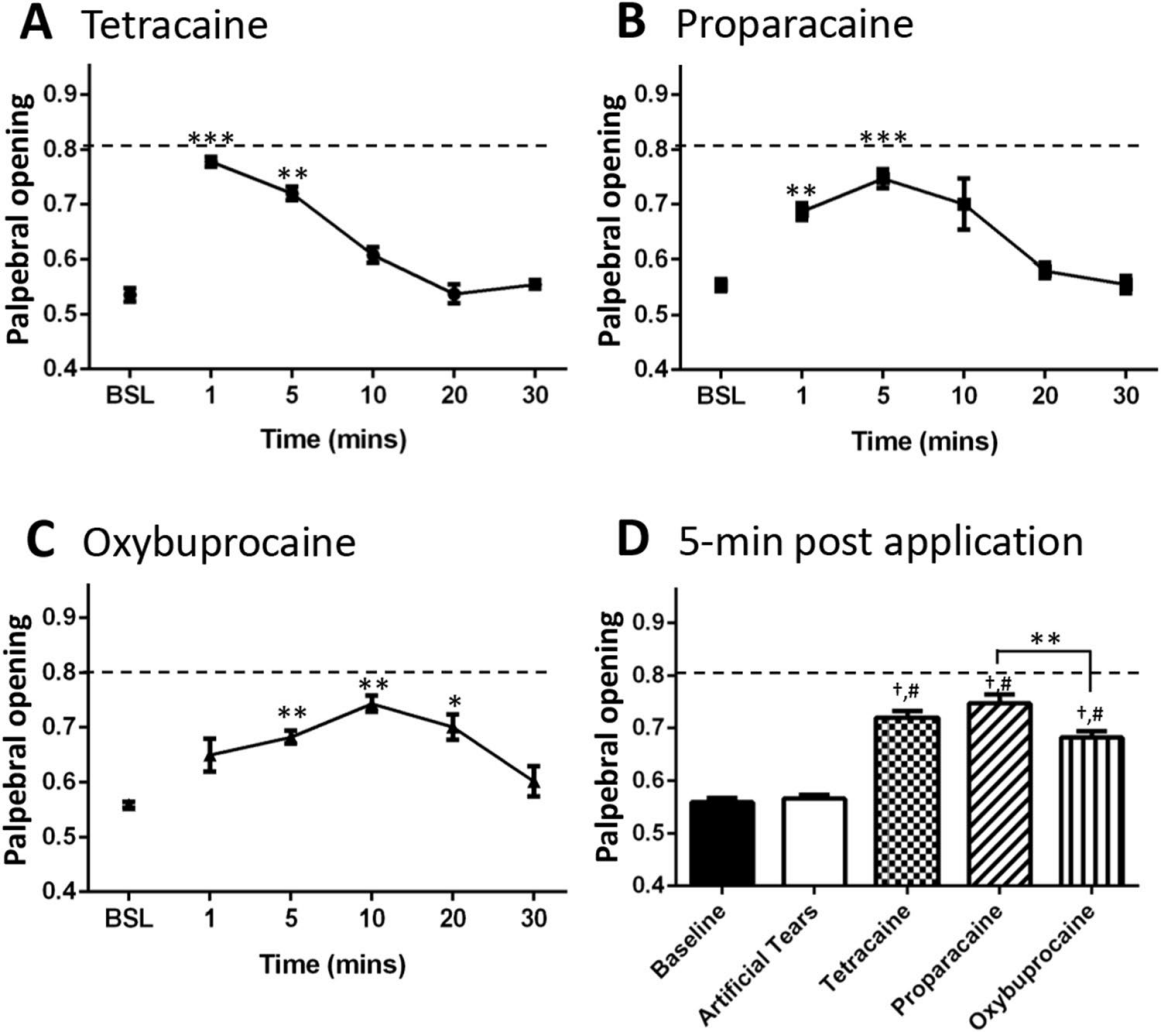
The effect of topical anesthetics on palpebral opening in double lacrimal gland excision treated animals. Corneal application of (**A**) tetracaine, and (**B**) proparacaine increased the palpebral opening for 5 min before gradually returning to baseline values. (**C**) Corneal application of oxybuprocaine produced a more prolonged anesthesia, with the palpebral opening returning to baseline after 30 min. (**D**) Corneal application of artificial tears had no effect on eye closure 5 min post application. BSL, baseline. n = 6 for each drug treatment group (3 females, 3 males). Dashed line represents naïve palpebral opening score. *p < 0.05, **p < 0.01, ***p < 0.001; ^#^p < 0. 0001 compared to baseline; ^†^p < 0.0001 compared to artificial tears.

The contribution of persistent corneal afferent activity in driving the decrease in palpebral opening was examined using the application of topical anesthetics to the eye. After taking a baseline measurement, palpebral opening was recorded following artificial tears or three commonly used corneal topical anesthetics. A significant effect of treatment was found for all three anesthetics (1-way ANOVA, Table 2), with each drug displaying a distinct time-course of action (Fig. 2). Tetracaine produced a peak effect on palpebral opening at one-minute post-application before rapidly declining (Fig. 2A), whereas proparacaine displayed a maximal effect after 5 min (Fig. 2B). For both compounds, palpebral opening was no longer elevated compared to baseline values within 10 min. In contrast, oxybuprocaine displayed a slower onset of action, producing a peak effect at 10 min post-application. Values remained elevated at the 20 min time point, giving oxybuprocaine the longest duration of action of the three compounds. Application of artificial tears did not affect palpebral opening when compared to baseline (Fig. 2D, Table 2).

**Table 2:** Results from one-way ANOVA statistical analysis.

Figure #	Measurement (units)	dfn, dfd	F	p
Figure 2A	Palpebral opening	5, 5	67.19	< 0.0001
Figure 2B	Palpebral opening	5, 5	15.08	< 0.0048
Figure 2C	Palpebral opening	5, 5	11.13	< 0.0065
Figure 2D	Palpebral opening	4, 27	60.25	< 0.0001
Figure 4A	Total Distance (cm)	2, 51	6.784	< 0.0024
Figure 4B	Total Distance (cm)	2, 47	5.146	< 0.0095
Figure 4C	Vertical rears	2, 51	5.179	< 0.0090
Figure 4D	Vertical rears	2, 47	4.419	< 0.0174
Figure 4E	Vertical rear Time (s)	2, 51	6.899	< 0.0022
Figure 4F	Vertical rear Time (s)	2, 47	5.719	< 0.0061

*Dfn* degrees of freedom numerator, *dfd* degrees of freedom denominator, *F* F statistic, *p* p-value.

### Sensitivity to corneal capsaicin following LGE

**Figure 3:**
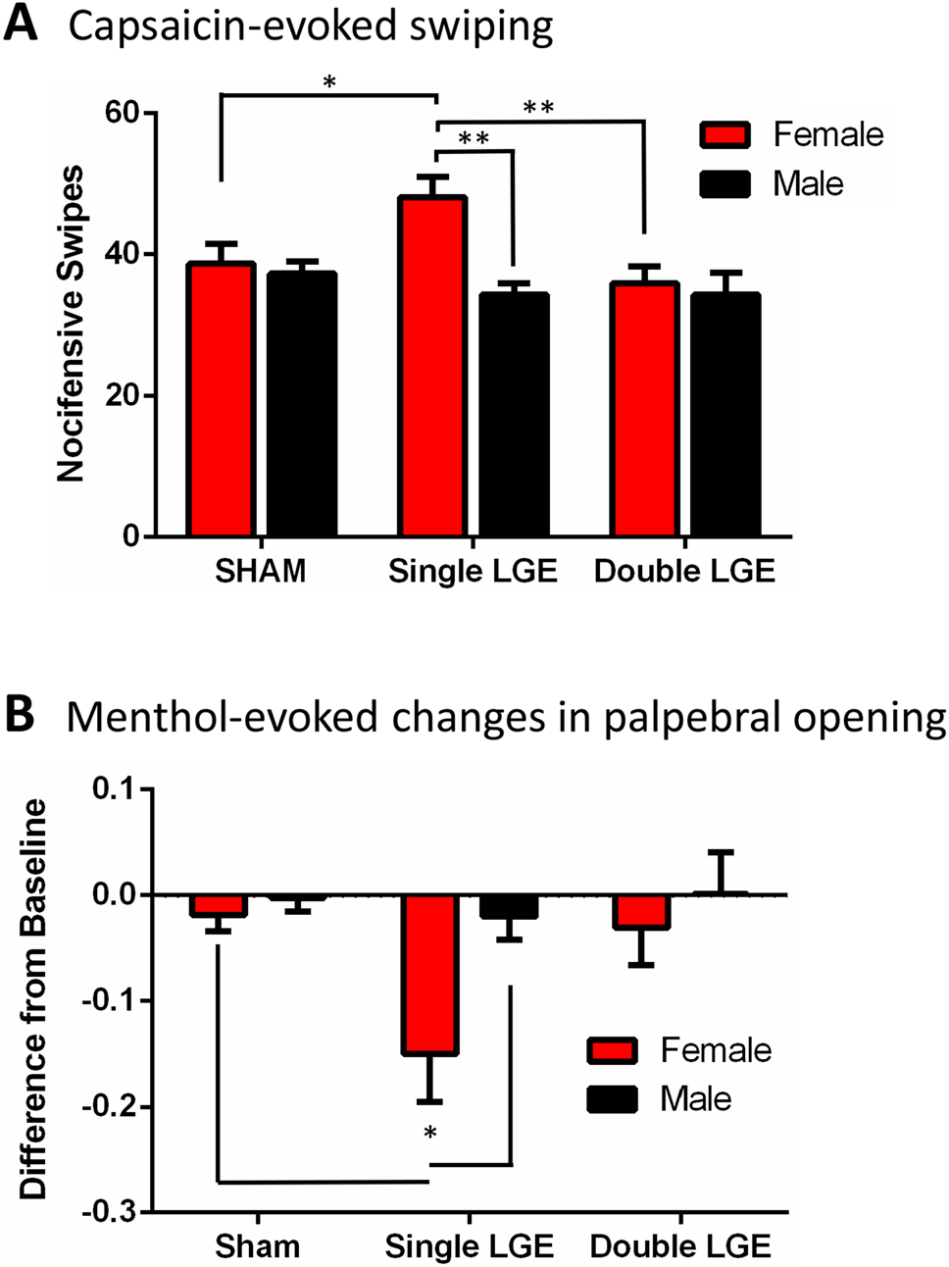
Ocular sensitivity to corneal application of capsaicin and menthol. (**A**) The number of nocifensive eye wipes after application of capsaicin was significantly greater only in female animals that had undergone single LGE. N = 11–12/treatment group. (**B**) After a baseline measurement, the palpebral opening was quantified 5-min post application of menthol. Menthol caused a decrease in the palpebral opening only in female mice with single LGE. n = 12/treatment group. *p < 0.05, **p < 0.01.

Eye wipe behaviors evoked by 0.1% capsaicin were used to examine the effect of LGE on the response to TRPV1 receptor activation 2-weeks after surgery. Following application of capsaicin to the eye, animals wiped using their forepaws, indicative of a nocifensive response^25^. A comparison of treatment group means found an overall significance of sex and surgery, along with a significant interaction between the two (2-way ANOVA, Table 1). Capsaicin evoked similar numbers of eye wipes in female and male sham treated mice. In female mice, however, capsaicin evoked a greater number of eye wipes after single LGE animals when compared to sham and double LGE treatment groups (Fig. 3A). Surprisingly, eye wipe behaviors after double LGE were no different from sham control animals. In male mice, neither single nor double LGE affected the number of capsaicin-evoked eye wipes (Fig. 3A).

### Sensitivity to corneal menthol following LGE

Previous studies have demonstrated LGE-induced sensitization of corneal cool cells to the TRMP8 agonist menthol^17^, yet the behavioral response to menthol after LGE has not been tested. Application of topical corneal menthol (100 and 200 μM) did not elicit an eye wipe response in either female or male animals after single or double LGE. An analysis of palpebral opening 5 min after menthol application indicated a significant effect of sex and surgery (2-way ANOVA, Table 1). Similar to the results with capsaicin-evoked eye wipes, a reduction in palpebral opening was found only in female mice after single LGE (Fig. 3B).

### Lacrimal gland excision reduces locomotion and rearing in the open field

**Figure 4:**
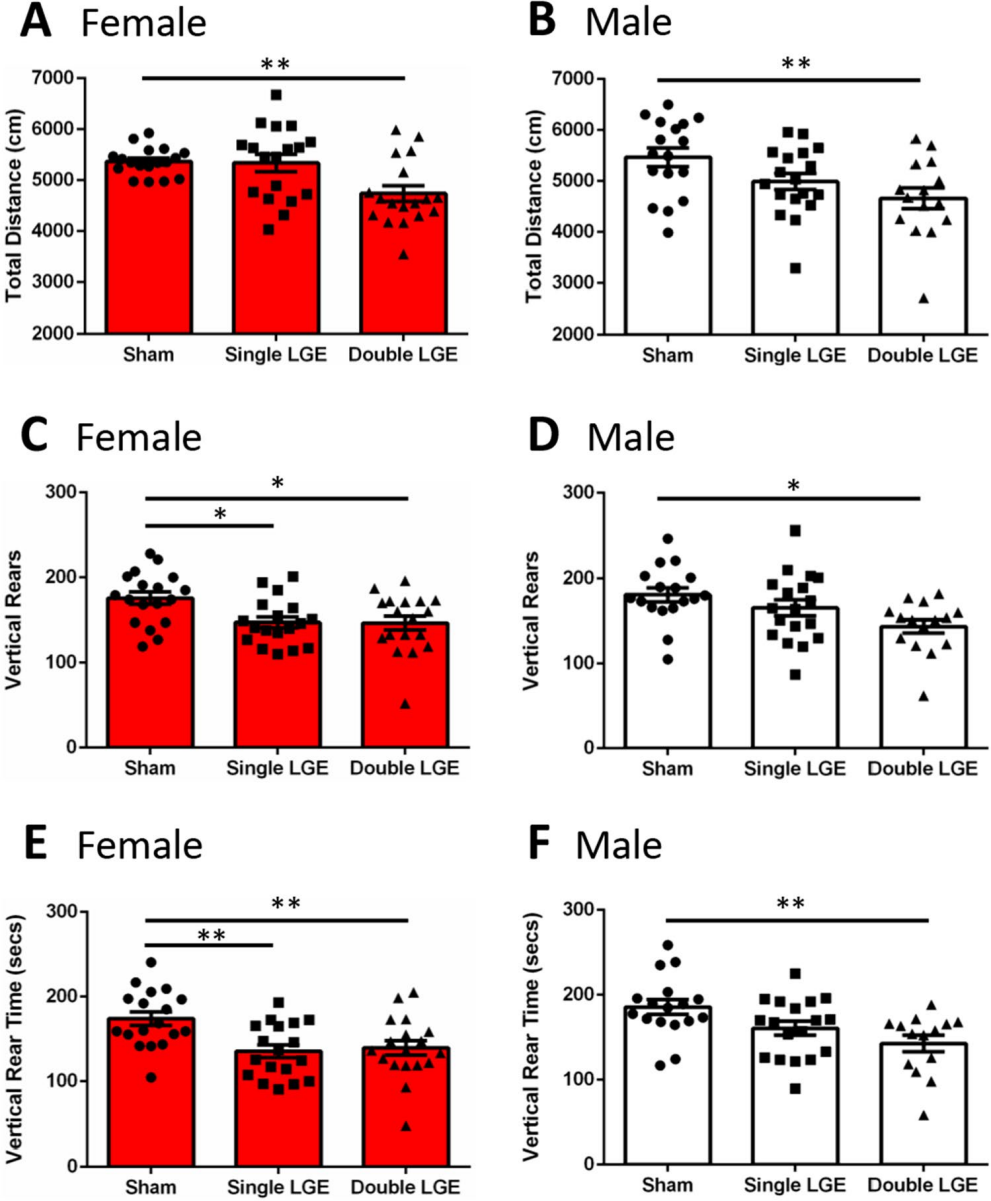
Locomotor activity following lacrimal gland excision. (**A**, **B**) Total distance traveled in female and male mice. Double LGE decreased the distance traveled in both female and male mice compared to sham treated controls. (**C**, **D**) Total number of vertical rears in female and male mice. In female mice, single and double LGE caused a significant reduction in total rears compared to sham controls. In male mice, only double LGE decreased the number of rears. (**E**, **F**) Total vertical rearing time for female and male mice. In female mice, single and double LGE caused a significant drop in vertical rearing time compared to sham controls. In male mice, only double LGE caused a significant drop in vertical rearing time compared to sham controls. n = 16–18/treatment group. *p < 0.05, **p < 0.01.

Ongoing pain has been demonstrated to effect open field behaviors, observed as a suppression of locomotor activity and a reduction in rearing behavior^26,27^. Exploratory behaviors were examined 2-weeks after sham, single LGE, or double LGE. A comparison of total distance traveled showed a significant effect of treatment in both female and male mice (1-way ANOVA, Table 2). Post-hoc analysis revealed a reduction in the total distance traveled after double LGE when compared to sham controls in male and female mice (Fig. 4A,B, p < 0.01).

A significant treatment effect was also found in the total number of rears and time spent rearing (1-way ANOVA, Table 2). In female mice, the number of rears was reduced after both single and double LGE when compared to sham controls (Fig. 4C, p < 0.05). Only double LGE reduced rearing in male mice (Fig. 4D, p < 0.05). Consistent with the reduction in number or rears, the time spent rearing was also reduced by single and double LGE in female mice (Fig. 4E, p < 0.01), and by only double LGE in male mice (Fig. 4F, p < 0.01). A comparison of male and female mice after sham surgery showed greater center time and movement in male compared with female mice, which was no longer apparent after LGE (see Supplemental Data).

### Lacrimal gland excision decreases open arm time in the elevated plus maze

**Figure 5:**
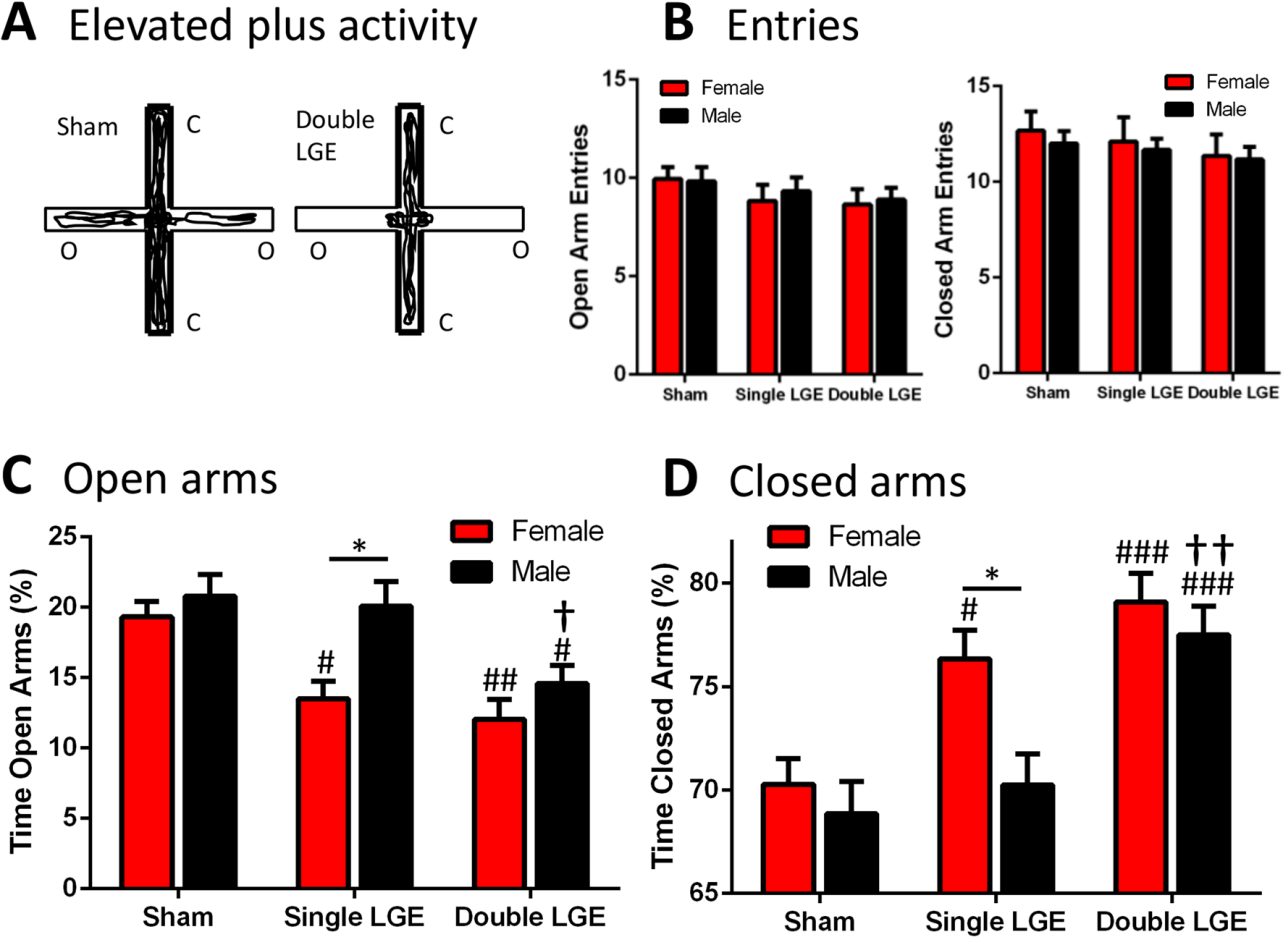
The effect of lacrimal gland excision on elevated plus maze behavior. (**A**) Representative examples of elevated plus maze activity in a sham mouse (left) and in a double LGE treated mouse (right). O, open arms; C, closed arms. (**B**) The number of open (left) and closed (right) arm entries were similar between treatment groups. (**C**) Percentage of time female and male mice spent in the open arms. Single and double LGE decreased the time female mice spent in the open arms compared to female sham animals. In male mice, only double LGE caused a significant decrease in open arm time. (**D**) Percentage of time female and male mice spent in the closed arms. Single LGE increased time in the closed arms only in female mice, whereas double LGE increased time spent in the closed arms in both female and male mice. n = 17–18/treatment group. *p < 0.05, **p < 0.01, ***p < 0.001; ^#^p < 0.05, ^##^p < 0.01, ^###^p < 0.001 compared to sham of the same sex; ^†^p < 0.05, ^††^p < 0.01 compared to single LGE of the same sex.

Reduced activity and rearing behavior in the open field are often indicative of increased anxiety^28,29^. The elevated plus maze was used to further examine the potential anxiogenic effects of LGE. Animals were free to explore the elevated plus maze apparatus for 5 min, as shown by the representative activity traces of a sham and double LGE treated mouse (Fig. 5A).

No effect of treatment or sex was found in the number of entries into the open and closed arms during the 5 min session (Fig. 5B, Table 1). A comparison between the different treatment groups of the time spent in the open arms and the time spent in the closed arms indicated a significant effect of both surgery and sex (2-way ANOVA, Table 1). For female mice, both single and double LGE significantly reduced the percentage of time spent in the open arms and increased the percentage of time spent in the closed arms compared to sham treated animals (Fig. 5C,D). For male mice, only double LGE caused a significant decrease in the percentage of time spent in the open arms, with a corresponding increase in the percentage of time spent in the closed arms (Fig. 5C,D).

### Corneal innervation

**Figure 6:**
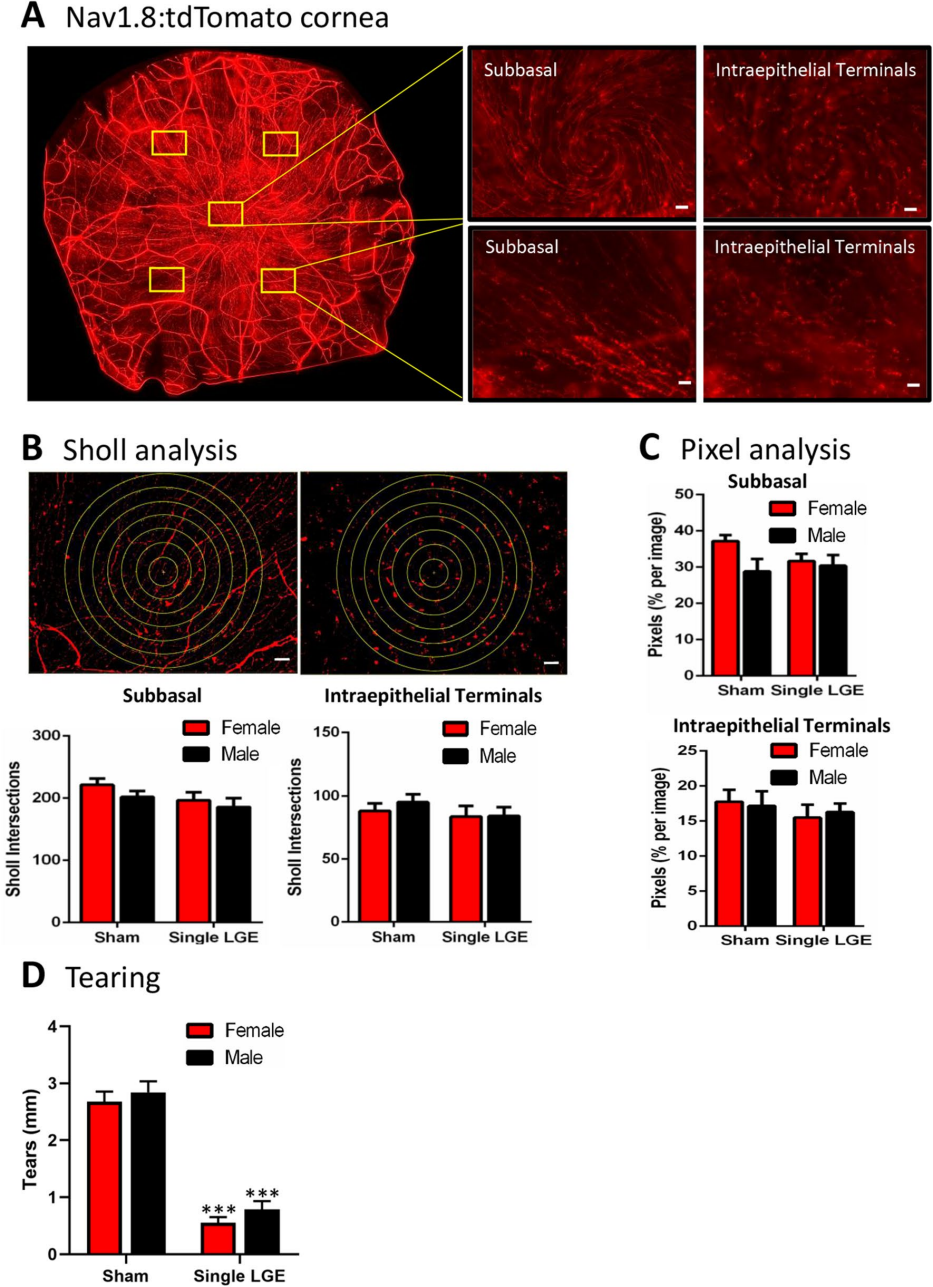
Corneal innervation two weeks after sham and single LGE in male and female Nav1.8-cre;tdTomato mice. (**A**) Representative image of the cornea showing nerve innervation for different regions of interest as well as layers including subbasal and intraepithelial terminals. Scale bar 20 μm. (**B**) Representative images showing the concentric rings placed onto the corneal regions of interest for use in the Sholl analysis for the subbasal nerve plexus (top left) and intraepithelial terminals (top right). Scale bar 20 μm. The number of Sholl intersections for the subbasal nerve plexus (bottom left) and intraepithelial terminals (bottom right) in female and male mice after sham and single LGE. (**C**) Pixel analysis of the subbasal nerve plexus (left) and intraepithelial terminals (right). (**D**) Tear measurements conducted 1-week after sham and single LGE. Single LGE reduced tear volume when compared to sham surgery in both female and male animals and no sex differences were observed. n = 6–8/treatment group. ***p < 0.001 versus sham operated controls.

Using Nav1.8-cre;tdTomato mice, corneal innervation was investigated and analyzed using Sholl and pixel analysis. Robust labeling of the subbasal nerve plexus and intraepithelial terminals were observed in the corneas of Nav1.8-cre;tdTomato mice (Fig. 6A). To quantify corneal nerve innervation, concentric rings were placed onto each image and averaged across the corneas for each group (Fig. 6B). At 2-weeks following sham and single LGE, there were no significant sex or treatment effects in the number of subbasal nerve or intraepithelial terminal Sholl intersections (Fig. 6B, Table 1). Using a trainable segmentation program, pixel quantification methodology showed a similar lack of sex or treatment effect (Fig. 6C). Of note, the single LGE in both female and male mice produced a highly significant reduction in tear measurements when compared to sham surgery (Fig. 6D, p  < 0.001, 2-way ANOVA, Table 1). Female and male mice had similar tear levels after both sham surgery and single LGE.

## Discussion

In a previous study, signs of corneal inflammation and epithelial cell damage were shown to be greater in female compared to male mice after the unilateral excision of either the extraorbital lacrimal gland (single LGE) or the extraorbital and intraorbital lacrimal glands (double LGE)^10^. In the present study, signs of ocular pain and anxiety related behaviors were examined two weeks after single and double LGE in male and female mice. Compared to male mice, single LGE in female mice produced greater signs of ocular pain and sensitivity, as measured using palpebral opening and reactions to corneal application of capsaicin and menthol. Likewise, single LGE in female mice increased anxiety related behaviors in the open field and plus maze. Double LGE induced comparable changes in corneal sensitivity and anxiety related behaviors in female and male mice. The increase in corneal sensitivity in female mice after single LGE was not accompanied by alterations in corneal nerve innervation density.

Persistent ocular pain after LGE has been previously explored in the rat after LGE by quantifying the rate of spontaneous eye blinks. Several of these studies have examined several time points, beginning one week after excision and extending out to 8 weeks. Lacrimal gland excision-induced sensitization of corneal polymodal nociceptors and cold cells occurred within one week after excision and persisted for at least 4 weeks^16–18^. In the present study, the 2-week time point was chosen because of the significant differences noted between female and male mice in corneal inflammation^10^. In male rats, a persistent increase in the rate of blinking has been reported over the course of up to 8 weeks after LGE^11,17,30–32^. A recent report comparing male and female rats found a greater increase in blink rate in female rats after both single and double LGE^13^. While rats demonstrate clear and easily quantifiable blinking behavior after LGE, mice displayed a consistent squinting of the eye ipsilateral to the excision rather than distinct eye blinks. In a previous study in rabbits palpebral opening was used to quantify eye closure after application of capsaicin^33^. A modification of this method was used recently to measure ongoing pain in female C57BL/6 mice after excision of the extraorbital lacrimal and harderian gland^8^. Similar to our findings, Fakih et al.^8^ reported a decrease in eye closure ratio after lacrimal and harderian gland excision.

Less severe dry eye, induced in the present study by extraorbital LGE, caused a reduction in palpebral opening only in female mice, which is consistent with the greater corneal damage observed in female compared to male mice 2-weeks after single LGE^10^. However, while double LGE produced greater signs of corneal damage and inflammation in female mice^10^, the reduction in palpebral opening was similar between females and males. This similarity in the reduction of palpebral opening, despite the reported differences in corneal damage, might not reflect different pain levels. Rather, palpebral opening may not convey the degree of pain in a graded fashion. It should be noted that one weakness in the present study is the absence of tear measurements and corneal damage assessments, thus preventing a direct correlation between these variables and the behavioral parameters measured. However, the large reduction in tearing quantified in the Nav1.8-cre;tdTomato mice after single LGE in both female and male mice is consistent with our previous study using this model^10^.

Previous studies have shown that unilateral corneal injury can produce bilateral effects on corneal innervation, immune cells, and cytokines^34–36^. For example, 14 days after unilateral trephine-induced corneal nerve injury, a contralateral reduction in stromal and sub-basal nerve innervation was found, along with bilateral ocular inflammation^36^. Corneal sensitivity in these studies, however, was not assessed. The reduction in palpebral opening after unilateral LGE was restricted to the side ipsilateral to the excision. This result is similar to the ipsilateral increase in blinking observed in the rat after LGE^11,15,32,37^ and suggests that unilateral LGE-induced corneal injury mainly affects ongoing sensitivity on the ipsilateral cornea. A systematic evaluation of the contralateral cornea after LGE that includes testing mechanical and chemical sensitivity has not yet been performed.

The effect of three different topical anesthetics on palpebral opening was examined, allowing for a comparison of anesthetic strength, onset, and duration. Tetracaine, proparacaine, and oxybuprocaine all have similar mechanisms of action, blocking voltage-gated sodium channels, with specific properties dependent on individual anesthetic affinities, hydrophobicity, and aromaticity which can account for the disparities in onset and duration of action^38^. A previous report in rats with LGE-induced dry eye found that proparacaine suppressed the blink rate, returning to baseline levels by 25 min after application^11^. Proparacaine’s duration of action on palpebral opening in the mouse was similar, peaking at 5 min after application with a return to baseline by 20–30 min. A comparison of the duration of action between tetracaine, proparacaine, and oxybruprocaine found that oxybuprocaine increased palpebral opening for the longest duration (20 min), although its onset appeared to be somewhat delayed. In contrast, tetracaine had a peak effect at 1 min post application, with palpebral opening returning to baseline values by 10 min. Previous studies comparing these three anesthetics have used ophthalmically normal animals and assessed corneal anesthesia using mechanically-evoked reflexes^39–42^. In these studies, tetracaine and oxybruprocaine had a similar duration of action, and tetracaine had a slightly longer duration of action when compared to proparacaine. In all cases, however, significant anesthesia was still present at 20 min, indicating that these compounds have a longer lasting anesthetic effect in ophthalmically normal eyes when compared to testing performed in dry eye animals.

The reduced effectiveness of topical analgesics in the dry eye animal could be the result of alterations in voltage gated sodium channels and potassium channels in the corneal afferents. Lacrimal gland excision in the guinea pig increased the excitability of corneal neurons, and whole cell patch clamp recordings revealed both increased sodium currents and decreased potassium currents in corneal cold neurons^16^. The shorter duration of action for these topical anesthetics also could be due to a low corneal pH environment caused by ocular inflammation, or to differences in the sensitivity of corneal afferents that drive the reduction in palpebral opening versus a mechanical-stimulation evoked response.

Eye wipe behaviors are a common nocifensive response that have been used to assess corneal sensitivity to noxious stimuli^43^. Previous studies have shown an increase in eye wipe behaviors following LGE in male rats in response to hypertonic saline and capsaicin^11,15^. In contrast to these results, Hegarty et al.^44^ found no changes in capsaicin eye wipe behaviors after lacrimal gland denervation in male rats. This result is similar to our findings in male mice 2-weeks post LGE. In female mice with single LGE, however, we found a greater capsaicin-evoked eye wipe response, suggesting the sensitization of TRPV1 expressing afferents. The increased nocifensive eye wipe behavior was not observed in male or female mice after more severe dry eye was produced by double LGE. This could be due to more extensive nerve damage and corneal denervation. It should be noted that corneal hypoesthesia has been reported in human subjects with dry eye even in the presence of ongoing irritation^45^.

Increased corneal sensitivity to capsaicin was observed despite the absence of morphological changes and innervation density in this treatment group. The cornea is the most densely innervated tissue in mammals, consisting entirely of unmyelinated C-fibers and lightly myelinated A-delta fibers^46^. To examine corneal innervation, the Nav1.8-cre;tdTomato reporter mouse line was utilized, since the voltage-gated sodium channel Nav1.8 is preferentially expressed in C-fibers, including > 90% of IB4-binding neurons (nonpeptidergic C-fibers) and CGRP-positive neurons (peptidergic C-fibers)^47^. Characterization of Nav1.8-cre;tdTomato mice showed robust corneal afferent neuronal labeling, including the subbasal nerve plexus and intraepithelial nerve terminals. Although decreased or aberrant nerve innervation has been found in some, but not all, studies of individuals with dry eye^48–52^, we found no significant difference in subbasal or intraepithelial nerve innervation for either sex after single LGE despite the significant reduction in tear volume. These results indicate that changes in corneal sensitivity can be observed without any detectable change in innervation.

In addition to increased eye wipe behavior, hypertonic saline and capsaicin-evoked orbicularis oculi muscle activity (OOemg) were increased in rats following LGE^15^. The hypertonic-saline evoked OOemg activity was blocked by TRPV1 antagonists and partially attenuated by a TRPM8 antagonist. TRPM8 has been implicated in dry eye pain^53^, yet TRPM8 activation has not been shown to elicit eye wipe behaviors in the rat^15^ or mouse^54^, including in the present study.

Corneal cold cells function to sense changes in ambient temperature and regulate basal tearing and blinking accordingly, without evoking nociceptive responses^54,55^. In tear deficient animals, corneal cold cells become sensitized to cooling^17,18,56^ and have been proposed to be responsible, at least in part, for the irritation or pain caused by dry eye^16^. The TRPM8 agonist menthol (200 µM) has been shown to increase OOemg activity to a similar degree in both control and LGE rats^15^, whereas 100 µM menthol had no effect in either group. In contrast, in the guinea pig 100 µM menthol increased blinking in LGE but not control animals, suggesting an increased sensitivity of the cornea to TRPM8 after LGE^16^. This result is consistent with our finding that single LGE decreased palpebral opening in female mice. The absence of any measurable change in palpebral opening after menthol application in double LGE animals is likely the result of a floor effect caused by the high degree of squinting produced by the LGE.

Ongoing pain has been assessed using several different paradigms, including observations of pain suppressed behaviors such as reductions in locomotor activity and wheel running^57^. The total distance traveled and number of rearings in the open field have been shown to be accurate indicators of pain intensity, comparable to reflex-based pain tests, and reversible with NSAID and morphine analgesics^26^. The suppression of total distance traveled and rearing behavior after LGE are consistent with these findings and could be indicative of ongoing ocular pain. It is also possible that the reduction in open field activity is related to an increase in anxiety, which is where the open field test is most often utilized^58,59^.

Pain is often accompanied, and can be exacerbated, by co-morbidities such as anxiety. Human studies have shown a positive correlation between DED severity and symptoms of anxiety^7,60–62^. While there have been some reports of increased signs of anxiety in chronic pain models, other reports have not found a significant effect of pain on anxiety measures^63–70^. Using the elevated plus maze, single LGE reduced open arm time only in female mice, whereas double LGE reduced open arm time in both female and male mice. The increase in anxiety in female mice after single LGE is consistent with their greater signs of ongoing pain when compared to male mice, as evidenced by a reduction in palpebral opening. It should be noted that visual acuity was not examined in the present study. While vision is unlikely to be affected after single LGE, as corneal innervation appears unchanged, it is possible severe dry eye induced by double LGE had an impact on visual acuity which could have affected anxiety related behaviors in the open field and plus maze. Future studies designed to examine the effect of anxiolytic and analgesic compounds on the open field and plus maze behaviors after LGE will help to determine whether dry eye-induced pain can be dissociated from the apparent signs of increased anxiety.

A unique feature of pain originating from the head and face region is the important role it plays alerting and protecting the organism from potentially life-threatening injury. The dense innervation of the cornea by nociceptors helps to protect the eye from damage, as the consequences of vision loss would be catastrophic to chances of survival. Consistent with its enhanced salience, studies have demonstrated greater fear responses elicited by trigeminal pain when compared to similar levels of pain in other parts of the body^24^. Anatomical findings support the vital role of trigeminal pain in evoking strong affective responses. Unlike dorsal root ganglion primary afferent neurons, a subgroup of trigeminal ganglion nociceptive neurons projects directly to the parabrachial nucleus, a primary relay for circuits involved in pain affect through connections to limbic regions including the amygdala, lateral hypothalamus, and bed nucleus stria terminalis^71^. These trigeminal pain-activated circuits may play a role in increasing anxiogenic behaviors in dry eye animals.

A compelling feature of dry eye disease is that it occurs significantly more in women compared to men. Researchers have identified a multitude of sex-related differences in the eye, which can be attributed to the actions of androgens and estrogens^22,72,73^. These sexually-dimorphic responses include hormone effects on the lacrimal gland, meibomian gland, cornea, and conjunctiva. Sex hormone receptor activation impacts aqueous tear output, lipid production, mucous secretion, and immune function^74–83^. In male and female mice, baseline tear volume measurements appeared similar and a comparable reduction in tears was produced after single LGE, consistent with our previous report^10^. Thus, hormonal influences on tear composition and/or immune responses after single LGE are likely to play a role in producing ocular conditions that support greater ongoing, persistent pain in female mice.

In summary, female mice were more susceptible to showing signs of corneal hypersensitivity and anxiety than male mice with moderate dry eye induced by single LGE. These findings are consistent with previous studies demonstrating greater corneal damage and inflammation in female mice after LGE^10^. Assessment of both pain and anxiety phenotypes after LGE may be useful in testing potential treatments for dry eye pain.

## Methods

### Animals

Male and female C57BL/6J mice aged 8–10 weeks were obtained from Jackson Laboratories (Bar Harbor, ME, USA). In order to examine corneal innervation following LGE, the Nav1.8-cre mouse generated by John Wood (University College London, UK) was generously provided by Sulayman D. Dib-Hajj (Yale University, New Haven, CT) with a C57/B6 background, rederived and bred with the reporter mouse B6.Cg-Gt(ROSA)26Sortm14(CAG-tdTomato)Hze/J (Jackson labs, stock #007914). These Nav1.8-cre;tdTomato mice exhibited robust labeling of corneal afferents, including stromal nerve bundles, subbasal nerve plexus, and intraepithelial terminals (Fig. 6A). Animals were housed in a controlled 12 h light/dark cycle with free access to food and water and treated according to the policies and recommendations of the National Institutes of Health guidelines for the handling and use of laboratory animals. All procedures were approved by the Institutional Animal Care and Use Committee at the University of New England.

### Surgical procedure

Surgeries were performed as previously described^10^. Briefly, under isoflurane anesthesia a unilateral excision or the lacrimal gland was performed, excising either the left extraorbital gland (single LGE), or both the extraorbital and intraorbital glands (double LGE). In this manner, the effects of a graded reduction in tears could be examined by comparing single LGE animals to double LGE animals^10^. For sham surgeries, incisions were made to partially expose both the extra- and intraorbital glands.

### Tear measurements

Tears were measured in unanesthetized Nav1.8-cre;tdTomato mice 1-week after single LGE or sham surgery by inserting cotton phenol red threads (Zone-Quick, FCI Ophthalmic, Pembroke MA, USA) into the lateral canthus of the eye for 20 s. The length of thread presenting with a change in color was measured under a microscope to the nearest 0.1 mm.

### Palpebral opening

As an indicator of ongoing irritation, eye closure (palpebral opening) was measured using a ratio consisting of the height of the gap between the upper and lower eyelids and the distance separating the two canthi. Mice were placed onto an elevated platform (4″ L × 4″ W × 3″ H) and allowed to habituate for 2 min. A video camera was mounted at the same height as the elevated platform and mice were recorded for 5 min following habituation. During video playback, snapshot photos were taken that showed the mouse eye perpendicular to the camera shot. Using ImageJ software, a measurement was taken to determine the distance between the upper and lower eyelid (y) as well as the distance separating the canthi (x). From these values, the y/x ratio was calculated to determine the palpebral opening.

Palpebral opening was examined after the corneal application of the TRPM8 agonist menthol (100–200 µM) and the topical anesthetics tetracaine hydrochloride, oxybuprocaine hydrochloride, and proparacaine hydrochloride. After taking baseline measurements, solutions were applied to the eye (10 µl) using a micropipette. Mice were placed on the raised platform and videotaped at 1-, 5-, 10-, 20-, and 30-min post-drug application.

### Eye wipe behavior

Eye wipe behavior was quantified after application of the TRPV1 agonist capsaicin and menthol. After pipetting 10 µl of 0.1% capsaicin or 200 µM menthol directly into the eye, animals were placed in a shallow dish and recorded for 2 min with a camera placed directly overhead. Videos were played back at 0.2 × speed for quantification of evoked wiping behavior. Eye wipes consisted of forepaw wiping directed toward the eye in which drug was applied. Normal facial grooming behavior was not included. No hind paw scratching was observed after capsaicin or menthol application.

### Locomotor activity

Locomotor activity and rearing behavior was measured in open field chambers (10.5″ L × 10.5″W chamber E63-12, Tru Scan Activity System, Coulbourn Instruments, MA, USA). Mice were acclimated to the room for one hour before being placed into the locomotor chamber for 15 min. Total distance traveled in the arena (cm), total time spent rearing, numbers of rears, center time (s), peripheral time (s), center distance (cm), and center entries was automatically calculated by the software calibrated for mouse settings.

### Elevated plus maze

The elevated plus maze apparatus consisted of two open arms (20″ L × 4.5″ W) and two enclosed arms (20″ L × 4.5″ W × 15.5″ H) elevated 20.5″ from the floor. The mouse was acclimated to the room for one hour before being placed into the center portion of the apparatus and recorded for 15 min by a video camera mounted above the apparatus. Time spent and entries in each arm was then manually quantified off-line.

### Drugs

Capsaicin solution was made by first adding 0.01 g of capsaicin (Sigma-Aldrich, MO, USA) to 150 μl of 100% ethanol. Separately, 850 μl of Tween 20 was mixed with 9.0 ml of artificial tears. The capsaicin/ethanol solution was then slowly added to the Tween 20/artificial tears while vortexing, giving a final solution of 0.1% (3.3 mM) capsaicin in 1.5% EtOH and 8.5% Tween 20^84–86^. Artificial tears consisted of 106.5 mM NaCl, 26.1 mM NaHCO_3_, 18.7 mM KCl, 1.0 mM MgCl_2_, 0.5 mM NaH_2_PO_4_, 1.1 mM CaCl_2_, 10 mM HEPES, pH 7.45. A stock solution of menthol (10 mM) was made from 0.156 g of menthol (Sigma-Aldrich, MO, USA) in 60 ml artificial tears and 40 ml of 100% ethanol. Working solutions of 100 and 200 µM were made by diluting the stock solution with artificial tears^16,17,87^. Tetracaine hydrochloride (USP 0.5%, NDC-24208-920-64, Bausch Lomb, NY, USA) and proparacaine hydrochloride (USP 0.5%, NDC-0404-7199-01, Henry Schein, NY, USA) were purchased as ophthalmic solutions. Oxybuprocaine hydrochloride (0.4%, B9050, Sigma-Aldrich, MO, USA) was dissolved in artificial tears.

### Tissue preparation

One, two, and four weeks after sham or single LGE, Nav1.8-cre;tdTomato mice were euthanized with Euthasol (NDC-051311-050-01, Virbac, TX, USA), followed immediately by enucleation of the left eye and fixation in 10% formalin for 15 min. Corneas were then excised along the corneal rim and quick fixed again for 15 min in formalin. The fixing process was followed by three ten-min washes in 0.1 M phosphate buffered saline to remove excess debris from the dissection. Whole corneas were mounted on a slide after cutting out two small pie slices, using a DAPI containing mounting medium.

### Image analysis

Corneal nerve photomicrographs were taken using a Keyence BZ-X700 microscope with a CFI Plan Fluor 40 × objective. Nerve fibers labeled with tdTomato fluorescent protein were viewed at an emission wavelength of 570–640 nm. Five areas of interest, one in the central cornea and four in the surrounding region, were imaged (Fig. 6A). Z-stack photos were taken at each area of interest to encompass both the subbasal nerve plexus and intraepithelial terminals. All images were acquired using the same exposure settings.

ImageJ software (version 2.0.0) was used to perform image analysis. Each z-stack was separated into two layers, one encompassing the subbasal nerve plexus and the other including only the intraepithelial terminals. Each stack was max projected using max intensity, followed by background subtraction. Auto thresholding using “Otsu dark” was utilized unless the threshold was calculated to be less than 10 (10,255). Each image was then converted to a mask and Sholl analysis was applied. Sholl analysis was used to quantify differences in the density of small diameter afferent neurons innervating the cornea. The intersections of nerve fibers and seven concentric circles, 100 µm apart, were counted with ImageJ (Fig. 6B). Using a trainable pixel segmentation program methodology showed the same results as the Sholl analysis (Fig. 6B,C).

### Statistics

After it was determined that data conformed to a normal distribution with equal variances, multiple group means were compared using either one- or two-way ANOVAs, with Tukey’s post hoc test if an overall significance was found (GraphPad Prism 6.01). Values are expressed as mean ± SEM, and p < 0.05 was considered to be statistically significant.

## Supplementary information

Supplementary Data.
