# Supplementary material for: Lacrimal gland excision in male and female mice causes ocular pain and anxiety-like behaviors
Source: Sci Rep. 2020 Oct 14;10:17225. doi: 10.1038/s41598-020-73945-w (PMC7560880; doi:10.1038/s41598-020-73945-w)
Supplement: Supplementary file 1 — Supplementary Data. [file 41598_2020_73945_MOESM1_ESM.docx]

**Lacrimal gland excision in male and female mice causes ocular pain and anxiety-like behaviors**

Neal E. Mecum^1,2^, Danielle Demers^1^, Cara E. Sullivan^1,3^, Tori E. Denis^1^, John R. Kalliel^1,4^, and Ian D. Meng^1,3,4 *^

^1^Center for Excellence in the Neuroscience, University of New England, Biddeford, ME 04005

^2^Molecular and Biomedical Sciences, University of Maine, Orono, ME, 04469

^3^Graduate Studies in Biomedical Sciences and Engineering, University of Maine, Orono, ME, 04469

^4^Department of Biomedical Sciences, College of Osteopathic Medicine, University of New England, Biddeford, ME 04005

**D.**





*

*

†

#





**C.**

*

#





**A.**

**

**

†

#





**B.**

*

#











**Supplemental Figure 1.** Locomotor activity following lacrimal gland excision. **A.** Center time, and **B.** Peripheral time in female and male mice. Sham treated male mice spent more time in the center and less time in the periphery compared to sham treated female mice. **C.** Center distance traveled, and center entries in female and male mice. Sham treated male mice traveled longer distances in the center and had more center entries than sham treated female mice. n = 16-18/treatment group. * p < 0.05, ** p < 0.01; # p<0.05 compared to sham of the same sex; † p<0.05 compared to single LGE of the same sex.
